# Drug-induced Sleep Endoscopy (DISE) with Simulation Bite to Predict the Success of Oral Appliance Therapy in Treating Obstructive Sleep Apnea/Hypopnea Syndrome (OSAHS)

**DOI:** 10.37825/2239-9747.1011

**Published:** 2020-10-01

**Authors:** Matteo Cavaliere, Pietro De Luca, Carla De Santis, Alfonso Scarpa, Massimo Ralli, Arianna Di Stadio, Pasquale Viola, Giuseppe Chiarella, Claudia Cassandro, Francesco Cassandro

**Affiliations:** 1 Otolaryngology Department, University Hospital “San Giovanni di Dio e Ruggi D’Aragona”, Salerno, Italy; 2 Department of Medicine, Surgery and Dentistry, University of Salerno, Salerno, Italy; 3 Department of Sense Organs, Sapienza University of Rome, Rome, Italy; 4 Department of Otolaryngology, University of Perugia, Italy Department of Otolaryngology University of Perugia Italy; 5 Department of Experimental and Clinical Medicine, Unit of Audiology and Phoniatrics, Magna Græcia University, Catanzaro, Italy; 6 Surgical Sceinces Department, University of Turin, Turin, ITaly; 7 Dentistry Unit, Department of Neurosciences, University “Federico II”, Napoli, Italy

**Keywords:** oral appliance, drug-induced sleep endoscopy, nasendoscopy, simulation bite, obstructive sleep apnea

## Abstract

**Study objectives:**

Oral appliances have gained their place in the treatment of obstructive sleep apnea (OSA) where custom-made titratable mandibular advancement devices (MAD) have become the oral appliance of choice. This study aimed to asses the value of the drug-induced sleep endoscopy (DISE) using a MAD in the prediction of treatment outcome for OSAHS

**Methods:**

This is a prospective, single-center cohort study that enrolled sixty-six consecutive patients with diagnosed OSA (5 events/h < apnea-hypopnea index (AHI) < 50 events/h) to be treated with a custom-made titratable MAD. The patients were evaluated polysomnographically with the MAD in situ after the adaptation and titration period of 3 months. The associations between findings during DISE and treatment outcome were assessed

**Results:**

The subjects showed a wide range of severity of OSAHS pre-treatment: median AHI was 43.10 with a range from 20.13 to 66.07. The simulation bite was associated with a significant increase in cross-sectional area at level of the velopharynx, tongue base and epiglottis. MAD treatment response in the studied population was 91%, with a mean AHI improving from 43.10 to 12.93.

**Conclusions:**

Drug-induced sleep endoscopy with simulation bite is an acceptably reproducible technique for determining the sites of obstruction in OSAHS subjects; it thus offers possibilities as a prognostic indicator for treatment with MAD

## I. INTRODUCTION

Obstructive sleep apnea/hypopnea syndrome (OSAHS) is characterized by recurrent episodes of apnea and hypopnea during sleep, due to repetitive upper airway (UA) collapse, often resulting in decreased oxygen blood levels and arousal from sleep [1,2]. Continuous positive airway pressure (cPAP) therapy is considered the gold standard for treatment, but oral appliance therapy could represent the main alternative treatment modality.

Mandibular advancement devices (MADs) are the most common class of oral appliances to treat OSAHS [3–5]; in selecting patients who might benefit from MAD treatment, several predictor factors of treatment outcome have been described in the literature, such as lower apnea–hypopnea index (AHI), lower body mass index (BMI), lower age, female gender and supine-dependent OSAHS [6,7]. Firstly described in 1991 by Croft and Pringle, sleep nasendoscopy or drug-induced sleep endoscopy (DISE) can be used to identify the level of snoring and UA collapse; a “chin-lift” manoeuvre (mandible actively guided forward by “grasping” it and advancing it to a maximal protruded position) can be added to the DISE procedure [8]. Some authors considered DISE a good technique for researchers to assess the site and the pattern of collapse, but they suggest that it should be correlated to clinically available measures, such awake nasendoscope to validate this.

The aim of this paper is to assess the value of the DISE using a MAD in the prediction of treatment outcome for OSAHS.

## II. METHODOLOGY

Sixty-six consecutive patients admitted to our Department with diagnosis of OSAHS were enrolled in the study. Sleeping Breath Disorders diagnosis was based on polysomnography (PSG)(ApneaLink Air. ResMed, Germany.). Parameters assessed were: AHI (Apnea Hypopnea Index) corresponding to average of apneas and hypopneas per hour of sleep; ODI (Oxygen Desaturation Index) which was expression of the number of desaturation events (> 3%) per hour of sleep; NADIR (the lower O_2_ saturation level).

OSAHS is defined by an AHI > 5h/sleep; baseline characteristics are shown in Table I.

Inclusion criteria were: >18 years of age, a confirmed diagnosis of OSAHS (AHI > 5h/sleep), prepared to wear a MAD, sufficient healthy teeth to allow MAD construction. We excluded patients with history of poorly controlled epilepsy, history of allergy to metals, edentulous or with insufficient teeth.

The study was approved by the Ethics Committee. All patients gave written consent.

### DISE with simulation bite

The technique of sleep nasendoscopy (SNE) was developed by Croft and Pringle in 1991: subjects are pharmacologically induced into a light phase of sleep, and the UA are directly visualized using a flexible fiber-optic endoscope. Levels of partial or complete obstruction are noted and the following grading scheme is applied: grade 1: palatal level snoring, grade 2: palatal level obstruction, grade 3: multi-segmental involvement – intermittent oro- and hypopharyngeal collapse, grade 4: sustained multi-level collapse, grade 5: tongue base obstruction [9]. An improvement of the nasendoscopic procedure has been to gently advance the mandible during sedation to mimic the effect of MAD and to examine the effect of this manoeuvre on UA patency and snoring: if airway dimensions improve and snoring is reduced, MAD may be indicated, regardless of the exact grading.

Artificial sleep was induced by intravenous administration propofol through a target-controlled infusion system in the operating room, with the patients in the supine position, with peripheral venous punction, monitoring oxygen saturation, cardiac rhythm and Bispectral Index (BIS). BIS monitoring provides direct information about the effects of anesthetics and sedatives on the brain by placing a sensor on a patient’s forehead [10,11]. The BIS monitor translates the information from electrical brain activity into a single number from 100 (indicating an awake patient) to 0 (indicating the absence of brain electrical activity). The search value during DISE ranged from 50 to 70.

When adequate sedation had been achieved, a nasolaryngoscope (Olympus ENF-GP, diameter 3.7 mm; Olympus Europe GmbH, Hamburg, Germany) was passed without local anaesthetic through the more patent nostril identifying the level(s) of any obstruction. Video-editing software was used to capture frames from the digital video recording of the nasolaryngoscopy.

All endoscopic findings have been classified according to the VOTE classification [12,13] which considers the following sites of obstruction: Velum, Oropharynx, Tongue base, Epiglottis. For each structure the minimal sectional area has been classified in 3 obstructing grades:

- grade 0: no obstruction;
- grade 1: partial obstruction;
- grade 2: complete obstruction.

Identification of the obstructing configuration was evaluated according to the shape of the dynamic collapse (antero-posterior, lateral or concentric when combination of the former two).

The DISE was preceded by the registration of a simulation bite for each patient. A customized, removable, Herbst appliance was created for each subject with the mandible in a position of maximum, comfortable protrusion. The patients were educated to protrude the mandible forward toward the maximal comfortable protrusion (MCP). We repeated three times and recorded this position.

Baseline DISE was complemented with the use of intra-orally simulation bite, placed by a dentist and preceded by intravenous administration of sedative drugs, in order to avoid jaw clamping or waking up the patient.

In the first phase of DISE we assessed the UA dimensions with the intra-orally simulation bite. The simulation bite was then removed, allowing assessment of the UA without any mandibular repositioning.

### Mandibular advanced device (MAD)

A custom-made mandibular advancement device (MAD) was fit for each patient; this consists of two dental clips (attached to each other) with an adjunct for adjust the manipular protrusion in the frontal teeth area. The patient needed three months to acclimatize with MAD; during this period, the adjunct was assessed to obtain a maximal comfortable protruded mandibular position or a resolute of the apneas.

### Evaluation

The patients were evaluated by an ambulatory polysonnography with the MAD in situ after the three-months adaptation and titration period. The associations between findings during DISE and treatment outcome were assessed. We considered as a treatment response a 50% reduction in AHI and an AHI value >20 [14].

## III. RESULTS

The subjects showed a wide range of severity of OSAHS pre-treatment: median AHI was 43.10 with a range from 20.13 to 66.07 (Table II).

Of sixty-six patients, the most suffered form severe OSAHS (45.4%), while seventeen patients (25.8%) and nineteen patients (28.8%) suffered from moderate and mild OSAHS respectively (Table III).

The DISE without simulation bite showed most frequently antero-posterior collapse at the level of the tongue base, a lateral collapse of the oropharynx lateral walls and a concentric collapse at the level of the palate. We found combined collapses in 65% of patients.

The simulation bite was associated with a significant increase in cross-sectional area at level of the velopharynx, tongue base and epiglottis. MAD treatment response in the studied population was 91%, with a mean AHI improving from 43.10 to 12.93 (Table IV).

The presence of palatal (concentric or antero-posterior) and tongue base antero-posterior collapse at baseline evaluation was associated with greater response to treatment, while the presence of oropharynx-lateral walls collapse at baseline evaluation showed a tendency towards an association with a less favourable treatment outcome (Table V).

## IV. DISCUSSION

OSAHS is defined as a disorder characterized by repeated collapse of the UA during sleep, with periodic apnea of breathing [15]. Depending on the severity of the condition, patients can be managed by nasally applied continuous positive airways pressure (nCPAP), mandibular advancement device (MAD) therapy or a range of surgical techniques [16,17].

Although less efficacious than CPAP, MADs are a simpler and often a more acceptable treatment modality, and it can represent a rescue therapy in patients with mild to severe OSA who are reluctant or non-responding to CPAP, or fail to use CPAP [18]. There has been increasing evidences about the use of MADs for the therapy of OSAHS and this treatment modality has been shown be more beneficial on polysomnographic indices of OSAHS, subjective and objective measures of sleepiness, blood pressure, neuropsychological functioning and quality of life.

Overall, ~60–70% of patients achieve a reduction of ≥50% in AHI, with a reduction of AHI to <5 events h^−1^ in 35–40% of patients [19–21].

MADs are worn intra-orally during the night and mechanically protrude the mandible, commonly with a design to gradually protrude the mandible applying a mechanical advancing mechanism, to prevent UA collapse and increase the cross-sectional airway dimensions, and thereby reduce snoring and obstructive sleep apneas [22]. Soft tissue structures within the palatoglossal and palatopharyngeal arches connect the mandible, tongue, lateral pharyngeal walls and soft palate; it has been proposed that such soft tissue connections may be stretched by mandibular advancement [23,24].

Using cephalometry, Tsuiki et al. [25] concluded that an anteriorly titrated mandibular position reduced obstructive sleep apnea severity, enlarged the velopharynx and decreased the curvature of the anterior velopharyngeal wall in good responders [5]. As the tongue is brought forwards, the soft palate will tend to follow to maintain a posterior oral seal, providing variable improvement in individuals with multi-level involvement [26]. This would explain the findings of Henke et al [27] that the MAD could be effective where the sites of obstruction include the velopharynx, and supports the more varied response observed here in subjects with multi-level involvement.

Several methods have been proposed to evaluate the effect of MAD on UA patency to predict its efficacy in the treatment of OSAHS. Although helpful in selected subjects, neither CT nor MRI can be justified routinely on the basis of expense, limited access and, in the case of CT, relatively high doses of radiation.

Anneclaire et al. [3] evaluated the associations between the findings during sleep nasendoscopy with simulation bite and treatment outcome. They reported a higher percentage of collapse at the level of the palate, followed by the tongue base and the epiglottis, while the oropharynx level was reported less commonly. There were several combinations of collapse levels, and the most frequent pattern was the combination of palatal and tongue base collapse (34.4%), while multi-level collapse in general was noted in 87.2% of all patients. In the present study, we reported a higher percentage of antero-posterior collapse at the level of the tongue base, a lateral collapse of the oropharynx lateral walls and a concentric collapse at the level of the palate.

When UA collapse was completely resolved using the simulation bite, the patient was considered a “well suitable” candidate for MAD treatment; patients with a partial resolution (residual collapse at one or more UA levels) were considered as “partially suitable”; patients were considered “not suitable” when UA collapsibility increased or remained unchanged with the simulation bite in situ.

They defined treatment response as a reduction in AHI following MAD treatment of ≥50% compared to baseline; overall MAD treatment response in the studied population was 68,9%, with a mean AHI improving from 21.4 to 8.9 h^1^ sleep. The presence of palatal collapse at baseline evaluation was associated with treatment response, while presence of hypopharyngeal collapse at baseline evaluation showed a tendency towards an association with a less favourable treatment outcome. The results of this study demonstrated a statistically significant association between a positive effect of the simulation bite on the UA patency during sleep nasendoscopy and treatment response with MAD (P < 0.01). We observed a positive response to treatment with MAD in 86.7% of antero-posterior velum collapse, 80% of concentric velum collapse, and 93.3% of antero-posterior tongue base collapse.

Ama et al. [28] evaluated the role of SNE in conjunction with mandibular protrusion to predict the success of MAD therapy in the treatment of patients with OSAHS; Chan et al [19] evaluated the role of MAD on UA anatomy using nasopharyngoscopy performed during wakefulness in patients with OSAHS and the potential role of this approach for identifying responder and nonresponder subjects, finding that the increase in the velopharyngeal cross-sectional area caused by mandibular advancement was significantly associated with a treatment response on polysomnography, while an increase in the cross-sectional area of the oropharynx or hypopharynx was not significantly associated with treatment outcome.

The results of our study suggest that the mandibular advancement manoeuvre during DISE could help to optimise the selection of patients for oral device treatment.

The major flaw of this study is that the assessment of the UA during sleep nasendoscopy is based on subjective findings.

## V. CONCLUSION

The development of a simple and clinically useful tool to predict which patients will respond to MAD therapy would aid the appropriate selection of patients. Drug-induced sleep endoscopy with simulation bite is a reproducible technique to assess the sites of obstruction in OSAHS patients, and it can be used as a prognostic indicator for treatment with MAD.

## Figures and Tables

**Table I:** Baseline characteristics of the study population.

*No. of patients*	66
*Age (years)*	49.59 ± 13.04
*Gender (M/F)*	60/6
*Body mass index*	29.87 ± 3.81
*Epworth Sleepiness Scale*	12.27 ± 3.98
**Polysomnographic study**	
*Apnea-Hypopnea Index*	43.10 ± 22.97
*Oxygen Desaturation Index*	38.30 ± 21.79
*Lower O* *_2_* * Saturation (%)*	75.82 ± 8.38
*% Time with O* *_2_* * Saturation <90%*	15.74 ± 16.40

**Table II:** OSAHS severity

OSAHS severity	N. Patients (%)
**Mild (5–14)**	19 (28.8%)
**Moderate (15–30)**	17 (25.8%)
**Severe(>30)**	30 (45.4%)

**Table III:** DISE endoscopic findings according to the VOTE classification.

Structure	Type of evaluation (with/without simulation bite)	Degree of obstruction	Configuration N. patients (%)		

			Antero-Posterior	Lateral	Concentric

Velum	*With*	***0.56 ± 0.49*** *	6 (9.1%)	4 (6.1%)	19 (28.8%)
	*Without*	***1.91 ± 0.29*** *	15 (22.7%)	6 (9.1%)	45 (68.2%)

Oropharynx – Lateral walls	*With*	0.91 ± 0.61		28 (42.4%)	
	*Without*	1.14 ± 0.89		45 (68.2%)	

Tongue Base	*With*	***0.33 ± 0.40*** *	8 (12.1%)		
	*Without*	***1.93 ± 0.42*** *	45 (68.2%)		

Epiglottis	*With*	***0.31 ± 0.39*** *	4 (6.1%)	3 (4.5%)	
	*Without*	***0.56 ± 0.43*** *	9 (13.6%)	6 (9.1%)	

* DISE with simulation bite vs DISE without simulation bite: p <0.05 (Student’s test)

**Table IV:** PSG Baseline versus MAD treatment

	Baseline	Evaluation with MAD after titration	P-value
AHI (mean ± SD)	43.10 ± 22.97	12.93 ± 11.62	***<0.001***
ODI (mean ± SD)	38.30 ± 21.79	11.49 ±10.34	***<0.001***

**Table V:** Response to treatment with MAD and DISE endoscopic findings

Structure	Response Type *	Configuration N. patients (%)		

		Antero-posterior	Lateral	Concentric

Velum	*Total N. per configuration*	15	6	45
	*Negative*	2 (13.3%)	3 (50%)	9 (20%)
	*Positive*	**13 (86.7%)**	3 (50%)	**36 (80%)**

Oropharynx – Lateral walls	*Total N. per configuration*		45	
	*Negative*		18 (40%)	
	*Positive*		27 (60%)	

Tongue Base	*Total N. per configuration*	45		
	*Negative*	3 (7%)		
	*Positive*	**42 (93.3%)**		

Epiglottis	*Total N. per configuration*	9	6	
	*Negative*	4 (44.5%)	3 (50%)	
	*Positive*	5 (55.5%)	3 (40%)	

* Response positive: a reduction in AHI following MAD treatment of 50% to baseline
